# Comparison of the molecular properties of retinitis pigmentosa P23H and N15S amino acid replacements in rhodopsin

**DOI:** 10.1371/journal.pone.0214639

**Published:** 2019-05-17

**Authors:** James Mitchell, Fernanda Balem, Kalyan Tirupula, David Man, Harpreet Kaur Dhiman, Naveena Yanamala, Julian Ollesch, Joan Planas-Iglesias, Barbara J. Jennings, Klaus Gerwert, Alessandro Iannaccone, Judith Klein-Seetharaman

**Affiliations:** 1 Division of Biomedical Sciences, Medical School, University of Warwick, Coventry, United Kingdom; 2 Department of Structural Biology, University of Pittsburgh School of Medicine, Pittsburgh, Pennsylvania, United States of America; 3 Department of Biophysics, Ruhr-University Bochum, Bochum, Germany; 4 Retinal Degeneration & Ophthalmic Genetics Service & Lions Visual Function Diagnostic Lab, Hamilton Eye Institute, Dept. Ophthalmology, University of Tennessee Health Science Center, Memphis, Tennessee, United States of America; Justus Liebig Universitat Giessen, GERMANY

## Abstract

Mutations in the *RHO* gene encoding for the visual pigment protein, rhodopsin, are among the most common cause of autosomal dominant retinitis pigmentosa (ADRP). Previous studies of ADRP mutations in different domains of rhodopsin have indicated that changes that lead to more instability in rhodopsin structure are responsible for more severe disease in patients. Here, we further test this hypothesis by comparing side-by-side and therefore quantitatively two *RHO* mutations, N15S and P23H, both located in the N-terminal intradiscal domain. The *in vitro* biochemical properties of these two rhodopsin proteins, expressed in stably transfected tetracycline-inducible HEK293S cells, their UV-visible absorption, their Fourier transform infrared, circular dichroism and Metarhodopsin II fluorescence spectroscopy properties were characterized. As compared to the severely impaired P23H molecular function, N15S is only slightly defective in structure and stability. We propose that the molecular basis for these structural differences lies in the greater distance of the N15 residue as compared to P23 with respect to the predicted rhodopsin folding core. As described previously for WT rhodopsin, addition of the cytoplasmic allosteric modulator chlorin e6 stabilizes especially the P23H protein, suggesting that chlorin e6 may be generally beneficial in the rescue of those ADRP rhodopsin proteins whose stability is affected by amino acid replacement.

## Introduction

Rhodopsin, the visual pigment of the rod photoreceptors, was the first identified genetic cause of retinitis pigmentosa (RP) disease and mutations in the rhodopsin gene (*RHO*) are the most common. Recent clinical studies support the conclusion that the two rhodopsin mutations under investigation in this study, P23H and N15S, yield distinct phenotypes in patients despite their close proximity in the rhodopsin structure [1]. In particular, the P23H mutation leads to greater and earlier *in vivo* rod function loss and an overall significantly greater disease severity than N15S. The aim of the present study was to investigate possible underlying molecular mechanisms for this clinical observation.

**Fig 1:**
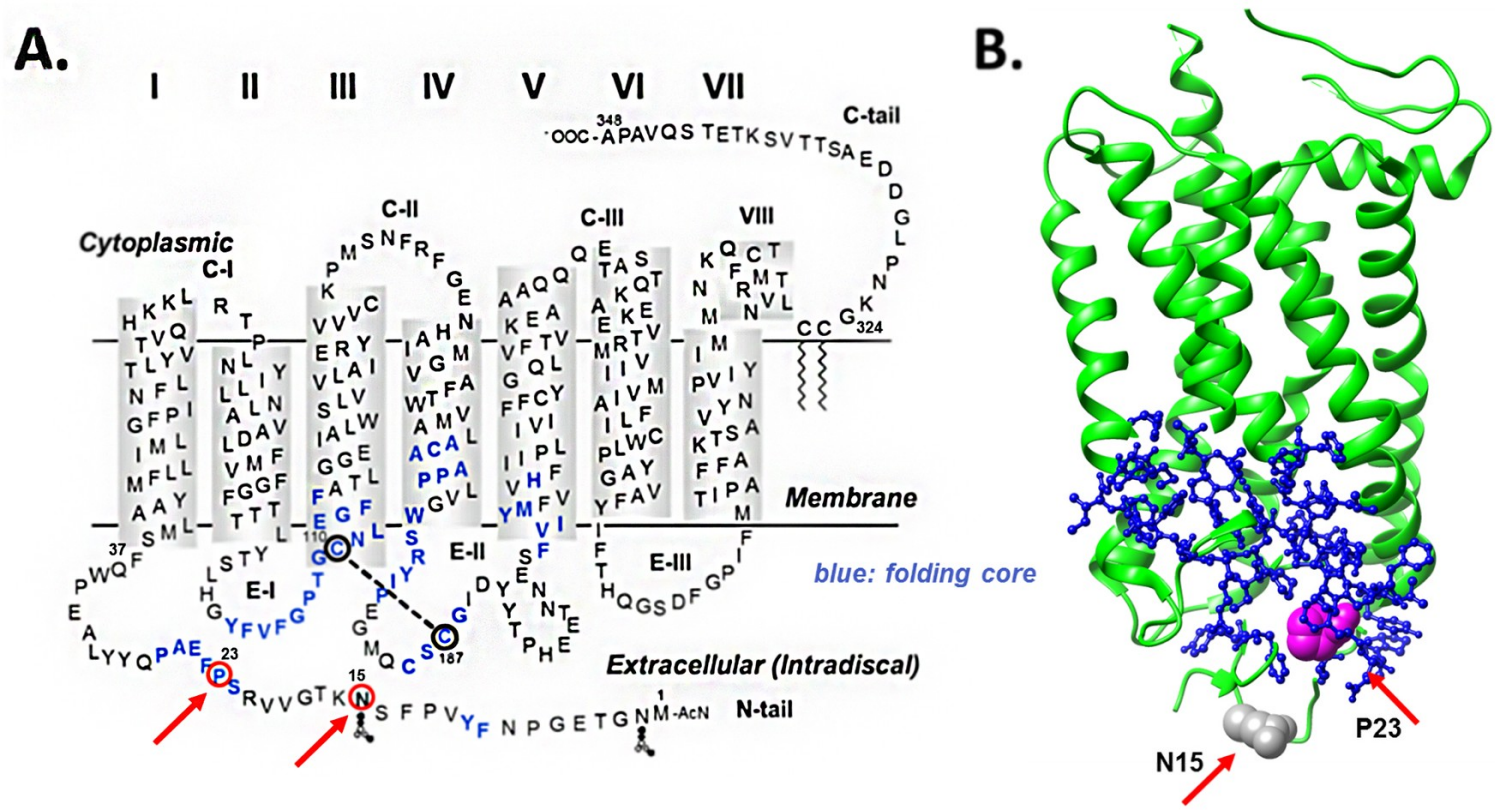
**A.** Secondary structure model of rhodopsin. The residues affected by the two mutations investigated in this manuscript, N15S and P23H, are shown encircled in **red** and **red** arrows. Predicted folding core residues based on FIRST analysis [48] are also highlighted **(blue)**. These are 9, 10, 22–27, 102–116, 166–171, 175–180, 185–188, 203–207, and 211. Based on these predictions, P23 participates directly in folding, whereas N15 does not. **B.** The relative folding role of the two residues is best appreciated in a 3D view of the tertiary structure of rhodopsin (1L9H) highlighting the **N15** (**grey**) and **P23 (magenta)** residues using space fill representation (**red** arrows). The 3D tertiary structure image is shown aligned with the secondary one with respect to its position relative to the discal membrane. Folding core residues (located intradiscally at the bottom of the molecule) are shown as ball-and-stick representation **(blue)**. Note the deep position of the P23 residue inside the folding core compared to the N15 residue, which sits below the core, further intradiscally.

In the *RHO* gene alone, there are 204 disease causing mutations according to the public version of the Human Gene Mutation Database (www.hgmd.cf.ac.uk). The molecular and clinical properties of many of these mutations have been reviewed in depth recently [2]. However, there are different schools of thoughts in terms of emphasizing the differences between mechanisms in the development of therapeutics. While some authors point out the diversity exhibited by the diverse mutations and the need to potentially address individual patients with different treatments [2,3], others draw generalities amongst disparate genetic causes, not only within a single gene (*RHO* in particular) but also across multiple genes associated with RP [4,5]. Further addressing this question is important because both, stabilizing and degrading unstable rhodopsin protein have been proposed as pharmacological interventions [2,5]. In the present investigation, we quantify the stability of secondary and tertiary structure of two point mutations associated with the autosomal dominant form of RP (ADRP), N15S and P23H. Both mutations are close in space (Fig 1) and affect the intradiscal domain at the N-terminus of rhodopsin.

P23H was historically the first change in the *RHO* gene associated with ADRP identified by Dryja and co-workers [6] and is one of the single most frequent mutations observed in ADRP patients in the United States of Northern European descent. When expressed recombinantly, the P23H protein yields much less chromophore with 11-*cis* retinal than wild type (WT), displays aberrant trafficking to the cell surface and its bleaching behavior is abnormal [3,7–9]. It is possible to overcome the low yield of folded P23H protein by addition of 9-*cis* or 11-*cis* retinal during expression *in vivo* [9–12]. This has enabled further characterization of this mutation in terms of reduced thermal stability of the dark and light-activated Metarhodopsin (Meta) II states, and reduced G protein activation [13,14].

N15 is one of the two glycosylation sites of rhodopsin [15]. Thus, the ADRP *RHO* N15S mutation compromises glycosylation at the N15 site [16]. Compromised glycosylation of rhodopsin at N2 even in the presence of normal glycosylation at position N15 has been proposed to be associated with extreme light sensitivity *in vivo*, a likely mechanism for the sectoral pattern of retinal degradation [17], and a decrease in Meta II stability [18]. On the other hand, the side-by-side comparison of N2S and N15S mutations in the *Xenopus* model system have suggested that it is the amino acid sequence at the extreme N-terminus rather than glycosylation of N2 itself that is the cause for retinal degeneration [3]. In the case of N15, on the other hand, it might actually be the lack of glycosylation itself that is responsible for the rod degenerative phenotype [3].

The thermal stability decay half-life of N15S at 37°C is 5.8 hours (WT: 58.3 hours, P23H: 1.7 hours) and 1.1 min at 55°C (WT: 72.5 min, P23H: 0.9 min), the Meta II decay half-life slightly higher than WT, and G protein activation is approximately 10% of WT [14]. Studies with another mutation at this site, N15Q, have indicated that impairments in this protein are not likely a result merely of the impairment in glycosylation [19]. Thus, more studies are needed to understand the molecular properties of mutations at position N15 [3].

Here, we present a quantitative comparison of the two mutants, which despite their location in the extracellular N-terminus show very different stability profiles. In particular, we find that the P23H mutation causes greater destabilization than the N15S one, a finding that parallels the differences in the clinical properties that we observed between these two mutations recently [1]. Although not proving causality, this observation does provide further support for the hypothesis that the molecular properties of the mutated rhodopsin proteins are both quantitatively and qualitatively linked to the phenotype observed in patients [20]. We show that adding the chlorophyll derivative, chlorin e6, shown previously to enhance the stability of WT rhodopsin [21–24], also stabilizes the P23H mutant, indicating that this molecular approach might be able to rescue the P23H phenotype.

## Materials and methods

For details please see the supplementary online methods file. 1D4 antibody was obtained from Dr. Robert Molday of the University of British Columbia, Vancouver, BC, Canada. 11-*cis* retinal was a gift from Rosalind Crouch, National Eye Institute, National Institutes of Health.

Single amino acid replacements P23H and N15S were prepared by a two-step PCR mutagenesis technique using the synthetic bovine opsin gene in the expression vector pMT4 [25]. The genes were then sub-cloned into the tetracycline inducible expression system vector pACMV-tetO as described [26], with the exception that we first disrupted the internal KpnI site and introduced a new KpnI site upstream of the start codon so that the genes could be introduced by KpnI/NotI restriction digestion and ligation. Tetracycline inducible HEK293S stable cell lines for P23H and N15S opsins were then established as described previously [26]. Transient transfection of COS-1 cells was carried out as described [25], with the exception that the cells were harvested 72h after transfection. Solubilization and purification by 1D4 immunoaffinity chromatography in 0.05% DM was carried out as described [27].

Purified proteins were subjected to recording by using a UV spectrophotometer (Perkin–Elmer λ 25). Thermal denaturation experiments were performed either at 55°C or at 37°C by following the loss of 500nm absorbance as a function of time, as indicated.

For cleavage of oligosaccharide chains from rhodopsin glycosylation sites, identical amounts of rhodopsin WT, P23H or N15S as judged by their absorbance spectra were reacted according to the manufacturer’s instructions with N-glycosidase F (PNGase F, Sigma) which cleaves the link between asparagine and N-acetylglucosamines. Images of immunoblots were used for densitometric analysis. The pixel density across lanes was extracted using imageJ [28]. The peaks caused by the ladder were automatically picked using in-house scripts written in R. A linear regression was performed on the log-values of the pixel position of each peak against the log-values of the theoretical MW. This regression was used to estimate a MW for each pixel value of blot profiles. Four blots were prepared in this way and collated for analysis. As the acquired images had regions in which the signal was saturated, each band was assumed, in the pixel density profile, to have a normal distribution. The background signal was corrected for by using a rolling disc geometric filter [29], then all values above 95% density for each lane were discarded as saturated. For most blots, the profile above 50 kDa was largely discarded in this way, so further analysis was limited to the region between 20 and 50 kDa. Gaussian peaks were then fitted by non-linear least squares to the remaining data, and the peak centres (in kDa) and share of the total area occupied by each was recorded. Boxplots summarising this information were constructed, with band centre in the y-axis and relative width of boxes representing fraction of total area occupied. To compare the region between 20 and 50 kDa with the region above 50 kDa, the areas under the curves were calculated using the trapezoidal rule and the fraction these areas occupied of the total area was compared between lanes.

Meta II fluorescence was measured as described [30] using a Varian Cary Eclipse instrument. In order to calculate Meta II half-lives, the data was analyzed by fitting to single and double component non-linear regression functions using Sigmaplot 10.0 scientific graphing software and R. While for WT a single half-life describes the data very well, both P23H and N15S, show deviations from a single exponential fit. However, while fitting to a two-component fit reproduced the data more accurately it also took more iterations to generate the fit and repeats of the experiment resulted in a larger spread in values obtained for each of the two components. We therefore decided to fit the data to a single exponential.

Fourier transform infrared (FTIR) spectra were collected for COS-1 cell rhodopsin reconstituted with 11-*cis* retinal and eluted from 1D4 sepharose in PBS/DM on a Bruker IFS 88 FTIR-spectrometer equipped with a 7 reflexion diamond μATR sensor (predecessor of DuraSamplIR II, Smiths Detection, UK) and the data was analyzed as described [31].

Circular dichroism (CD) spectra were acquired using a JASCO J-810 spectropolarimeter with samples of rhodopsin and Ce6 at 2.5 and 100°μM concentrations, respectively as described [21]. Qualitative estimates of helix content were obtained from the CD spectra by spectral deconvolution using CDPro software [32,33]. Protein reference set 10, which includes membrane proteins, was used.

## Results

### Estimation of the degrees of misfolding in N15S and P23H rhodopsins

**Fig 2:**
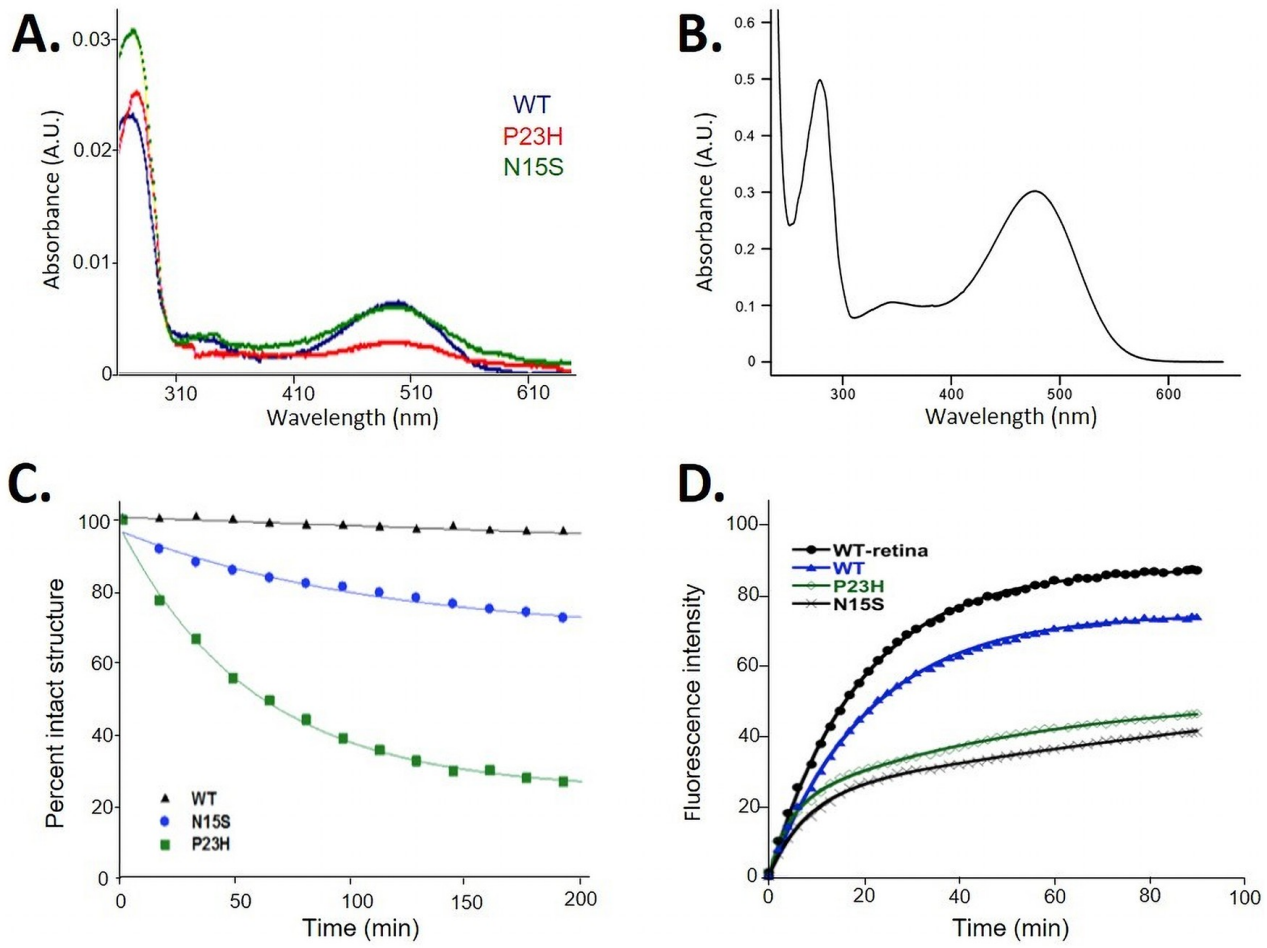
**A**. Overlay of absorbance spectra of rhodopsin proteins expressed in COS-1 cells and purified using 1D4-affinity chromatography. Proteins were eluted in phosphate buffered saline in order to elute both folded and misfolded portions. **B.** P23H rhodopsin obtained from stable cell line P23H and eluted in 2 mM sodium phosphate pH 6, which only elutes the folded fraction. **C**. Rhodopsin stability measured by loss of 500 nm chromophore over time at 37°C. Absorbance spectra of WT, N15S and P23H rhodopsin expressed in the presence of 9-cis retinal at 37°C were measured over time, and the decrease in absorbance at 500 nm was expressed at percent of intact structure. **D.** Rhodopsin Meta-II decay monitored by the use of fluorescence spectroscopy. Fluorescence spectra of rhodopsin Meta-II decay of WT, N15S and P23H rhodopsin expressed in the presence of 9-*cis* retinal.

Misfolding of rhodopsin is characterized by the inability of opsin to bind 11-*cis* retinal and is therefore detectable by increased absorbance ratios A_280_:A_500_ of rhodopsin purified in the presence of high concentrations of salt and high pH, such as phosphate buffered saline (PBS) solution. This is due to the A_280_ increased caused by increased opsin protein concentrations without concomitant retinal absorbance at A_500_ [34]. Thus, the ratio is an indirect measure of the fraction of misfolded protein, where a ratio of ~1.6 indicates 100% of molecules are correctly folded. The absorbance spectra of the three proteins in PBS is shown in Fig 2A. The A_280_:A_500_ absorbance ratios were 1.9±0.8, 13.4±2.6 and 5.7±0.3 for WT, P23H and N15S, respectively, indicating that P23H had a lower yield of correctly folded rhodopsin than N15S.

### Rescue of chromophore in N15S and P23H rhodopsins by the addition of retinal

Misfolded P23H rhodopsin can be pharmacologically rescued by addition of 11-*cis* or 9-*cis* retinal to cell cultures [9–11]. We made use of this observation by adding 9-*cis* retinal to cell cultures during expression. Furthermore, in order to obtain larger quantities of proteins, we created stable HEK293 cells of the two mutant proteins. Purification of rhodopsin from either plates or spinner flasks of our stable cell lines yielded WT like amounts on the order of 2mg/liter culture. Elution fractions from 1D4 sepharose now yield ratios of 1.6, indicating that all opsin has bound retinal, as shown in the example in Fig 2B for P23H.

### Rhodopsin stability measured by loss of 500nm chromophore over time

The thermal stability of N15S and P23H rhodopsin proteins in comparison to WT purified from HEK293 cells expressing rhodopsin in the presence of retinal is illustrated in Fig 2C. At this temperature, there is no loss in chromophore for WT rhodopsin. The thermal stability of N15S was ~25% less as compared to that of WT at 200 min, whereas P23H was highly unstable (~75% less intact structure at 200 min), which is in agreement with previous studies where P23H was also less thermally stable and presented lower photosensitivity than the WT [13]. The average half-life [±standard deviation (SD)] of the N15S and P23H were 101.0±27.6 min and 52.6±13.1 min, respectively. Similar results were observed at higher temperature (55°C), when rhodopsin transiently expressed and reconstituted from COS-1 cells (data not shown). These results demonstrate that even though the expression level and folding improved upon addition of 9-*cis* retinal during expression, the purified proteins show the same stability pattern regardless of the source. Both proteins are less stable than WT, with P23H being much less stable than N15S.

### Stability of retinal-opsin interactions after light-activation at 20°C

The difference in stability after light activation was quantified by fluorescence spectroscopy measuring the decay of light-activated rhodopsin Meta II to free retinal and opsin (Meta II decay) at 20°C based on intrinsic fluorescence of the five tryptophan residues in rhodopsin—see Fig 1 for their location—using published protocols [30]. If the protein is less stable, it decays faster, which can be expressed in a decreased half-life (*t*_*1/2*_) of the Meta II species. The fluorescence traces are shown in Fig 2D. Total fluorescence after light activation was lower than WT for both P23H and N15S, possibly due to aggregation which can quench tryptophan fluorescence. The estimated *t*_*1/2*_ values (±SD) for WT purified from bovine retina (n = 2), for WT purified from stably transfected HEK293S cells (n = 3), P23H and N15S purified from the respective stable HEK293S cell lines (n = 4 each) were 13.3±0.3 min, 14.5±0.2 min, 12.5±0.5 and 11.4±1.0 min, respectively. The data indicates that overall the deviation of *t*_*1/2*_ values from WT, regardless of source (bovine retinae or cell lines) are relatively small, similar to what has been observed previously [13,14]. There is a slight reduction in Meta II stability when going from WT to N15S and further to P23H, based on their progressive decrease in *t*_*1/2*_ values. However, the total fluorescence signal was decreased for both N15S and P23H, indicating a difference in the properties of the opsin formed after retinal leaves the binding pocket, highlighting the pathogenic properties of both mutants as compared to WT.

### Glycosylation and aggregation of WT, N15S and P23H in COS-1 cells

The decreased total tryptophan fluorescence in Meta II assays indicated that both P23H and N15S proteins may be more prone to aggregation than WT (Fig 2). Misfolding and aggregation also lead to aberrant glycosylation patterns for P23H [19] and the N15S mutation directly impairs glycosylation at the N15 site. To study both phenomena, aggregation and glycosylation, directly comparing P23H and N15S, we conducted PNGase F treatment of the purified proteins, followed by SDS-PAGE and Western blot. Since we purified the proteins and quantified them by absorbance spectroscopy, we were able to load identical amounts without the need for loading controls.

**Fig 3:**
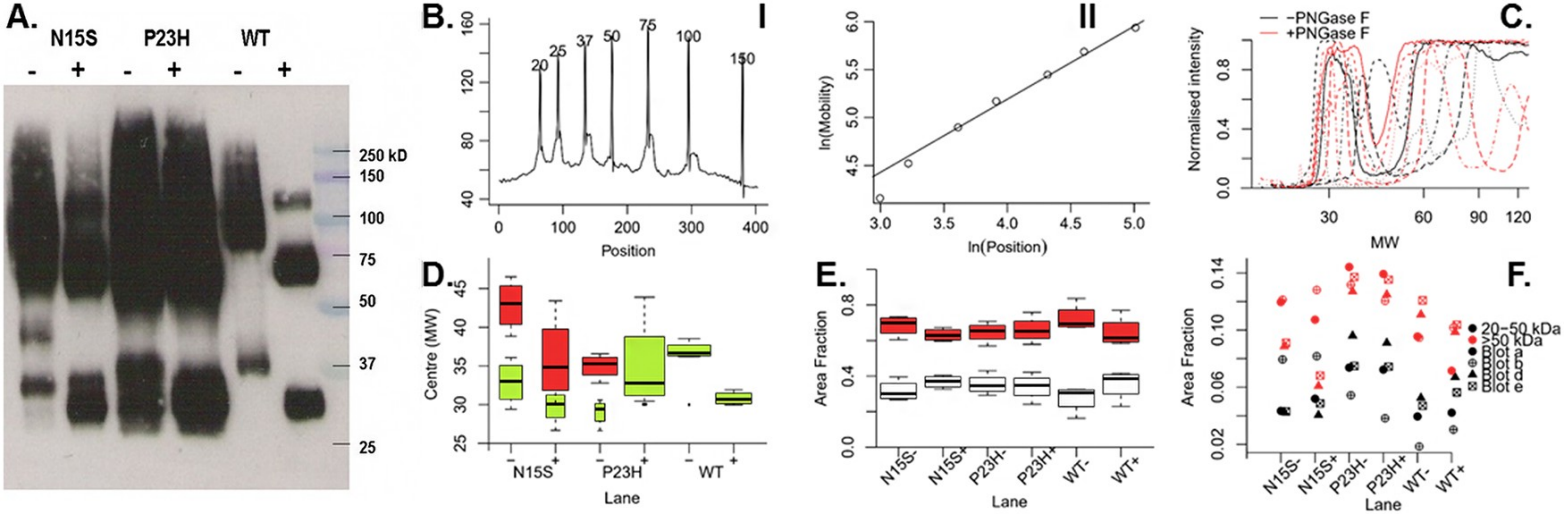
Deglycosylation analysis of rhodopsin. **A.** Example blot with rhodopsin mutation (N15S, P23H and WT transiently in COS-1 cells), and PNGase F treatment (+/-) by lane indicated. Protein concentrations were adjusted according to expression level and approximately 1μg was loaded. **B.** Use of the ladder to obtain molecular weights for mobility values for the blot shown in A. **(I)** Peak picking was performed automatically by selecting the local maxima. **(II)** Linear regression performed on ln(MW) and ln(Position). This was then rearranged so molecular weights could be estimated for each pixel. **C.** Density profiles aligned by the process in B. for WT rhodopsin. **Black** and **red** lines show profiles before and after PNGase F treatment respectively. **D.** Boxplots of centres of peaks fitted to profiles (e.g. C.). Box widths are proportional to the relative area occupied by that peak in the region from 20 to 50 kDa. **E.** Boxplots of the area fraction of density profiles separated by lane from numerical integration between 20 and 50kDa (**white**) and above 50kDa (**red**). **F.** Scatterplot of the area fraction of the density profiles of all lanes in a gel from numerical integration of the categories as in E.

The results of the Western blot analysis are shown in Fig 3E and 3F and **Table A in** S1 File for rhodopsin expressed in COS-1 cells, in the absence of retinal addition. It is well known that rhodopsin expressed in COS-1 cells displays heterogenous levels of glycosylation which is seen in diffuse bands, contrasting to the sharp bands observed in rhodopsin in rod outer segments [19]. Therefore, rhodopsin displays diffuse bands corresponding to monomeric and dimeric rhodopsin before PNGase F (–) treatment due to this heterogeneous glycosylation. Distinct bands at lower molecular weights were observed after the treatment (+), because now the bands correspond to protein only and the cause for heterogeneity (sugar moieties) has been removed.

In WT(-) there is one band at 37kD (see **Table A in** S1 File), the glycosylated monomer, and one broad band at ~80kD and above, the glycosylated dimer. After PNGase F treatment WT(+), there is a distinct band at 31kD (protein only monomer) and at ~65kD (protein only dimer), and a minor band at ~120kD (tetramer). Both, P23H and N15S, differ distinctly from WT, albeit in different ways.

In N15S(-), one glycosylation site is missing, causing a shift in molecular weight of the monomer species to lower molecular weight than WT, at 33kD (**Table A in** S1 File). In addition, there is evidence of fully unglycosylated species. The lowest molecular weight band is a band of very low intensity at the same molecular weight as WT(+), indicating that this band corresponds to a small fraction of fully non-glycosylated species. Slightly above is the main monomeric band around 33kD, corresponding to a single glycosylation site, which is why this band is below the WT rhodopsin monomer band at 37kD. This pattern has been observed previously in the T17M RP mutant that is also known to lack glycosylation at N15 [3]. The next band in N15S(-) is observed around 43kD, above the monomer but below the dimer molecular weights. Like the other bands, this band is highly reproducible and has been observed every time we have prepared this protein and run a gel. This band was not seen in the T17M mutant [3], but we have overexposed our blot in contrast to the very weak intensity in the T17M blot, which may mask the visibility of this species. There is, however, evidence for this species in the related N15Q opsin [19]. While we are not certain at this stage what this band corresponds to, it is tempting to speculate that this corresponds to an over-glycosylated monomer species, rather than a degradation product of the dimer. This is because this band disappears upon PNGase F treatment without corresponding appearance of a new band below the deglycosylated monomer, which is what would be expected in the case of a degradation product. Finally, at the dimer positions, the N15S(-) bands likely correspond to unglycosylated dimer and monoglycosylated dimer. After PNGase F treatment (+), the N15S protein appeared very similar to WT, demonstrating a relatively mild phenotype of N15S rhodopsin also at the molecular level.

In the case of P23H, the proportion of unglycosylated species was much higher than in N15S (and which is completely absent in WT), with glycosylated (–) and non-glycosylated (+) monomeric species being similar in intensity. There was also intensity at lower molecular weights for both the monomer and dimer bands, indicating that there is also some degradation occurring for P23H. Degradation of P23H has been studied extensively before, as reviewed in [2].

For both, P23H and N15S, the intensities of dimer (~65kD) and higher (>100kD) molecular weight were much higher than in WT, indicating that there was strong evidence of aggregation. Aggregation was higher in P23H than in N15S which showed very high intensity at 100kD and above.

Qualitative analysis of the Western blot data suggest that both the degree of aggregation and aberrant glycosylation in P23H were more severe than that of N15S. We quantified the relative band intensity in three independent experiments through automated peak selection with no prior assumption on number or positions of peaks (see Methods). The results are shown in Fig 3F for the range in the gel corresponding to monomer species. In the WT, there are two distinct bands, corresponding to glycosylated (-) and unglycosylated (+) monomer species, with very little spread in their molecular weights. In contrast, both mutants show several broad distributions in the same weight range (shown in red in Fig 3F). The patterns for the monomeric bands of WT, P23H and N15S differ from each other, clearly showing differences in glycosylation states.

### FTIR spectroscopic analysis of WT, N15S and P23H rhodopsin proteins

**Fig 4:**
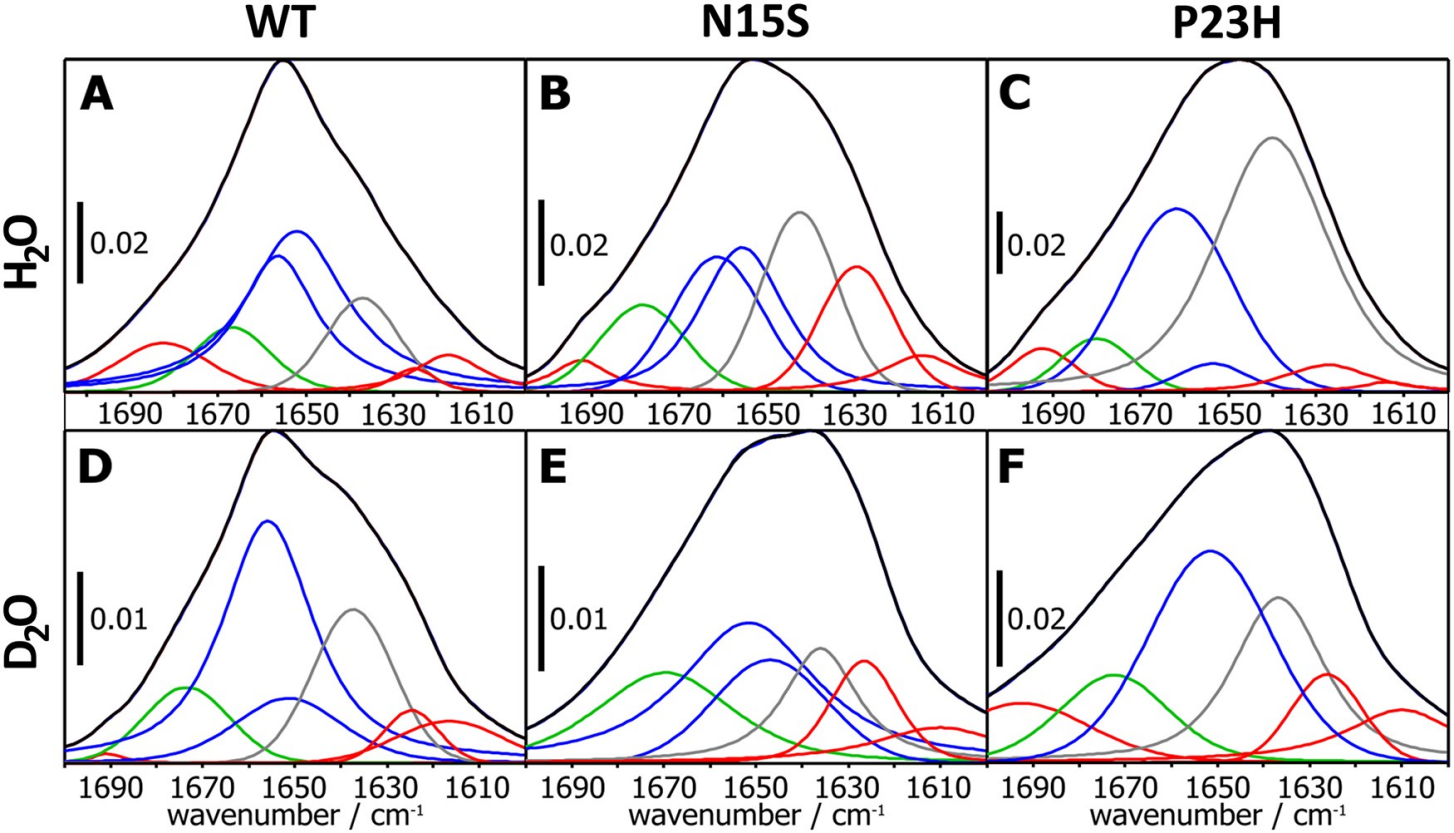
The FTIR amide I band region along with the deconvoluted spectra corresponds to different secondary structure fractions of the three samples. A-C: Rhodopsin in H_2_0, D-F: Rhodopsin in D_2_0: A, D: WT; B, E: N15S; and C, F: P23H. Bands assigned to β-sheets are indicated **red**, β-turns **green**, α-helices **blue**, and random coil **grey**.

To gain structural insight into the extent and molecular nature of misfolding in the two mutant proteins compared to the WT protein, we used FTIR spectroscopy. The amide I region of the spectrum corresponds mainly to C = O stretching of the peptide backbone and thus reports on secondary structure elements. The spectra are shown in Fig 4 and the relative intensities of α-helix, β-sheet, random coil and turns are listed in Table 1. Overall, the amide I bands (**black** lines in Fig 4) of N15S and P23H appear broader as compared to WT rhodopsin. In WT, the content of the observed estimates are in good agreement with the estimated secondary structure content from the X-ray crystal structure of rhodopsin (9% root mean squared deviation). In the case of N15S rhodopsin, a decrease in α-helix content (42%), and an increase in β-sheet (25%), random coil (21%) and turns (12%) was observed when compared to WT. The P23H rhodopsin exhibited even more severe structural defects when compared to WT: a strong increase in the random coil fraction (52%) was observed, and the α-helix, β-sheet, and turn fractions decreased to 32%, 10%, and 6%, respectively. These results indicate that the structure of N15S is more folded than the P23H rhodopsin. Similar results were obtained when the samples were exchanged to D_2_O, which leads to less overlap in different secondary structure elements).

**Table 1:** Secondary structure composition of WT, N15S and P23H before and after H/D exchange as obtained from FTIR spectra (Fig 4). The root mean squared deviation of both the H_2_O and D_2_O datasets from x-ray data estimates 9% total uncertainty.

Secondary Structure Details (in %)	STRIDE	WT_PBS		N15S_PBS		P23H_PBS	
	PDB 1L9H	H_2_0	D_2_0	H_2_0	D_2_0	H_2_0	D_2_0
**α-Helix**	64	63	59	42	48	32	33
**β-sheet**	3	16	12	25	18	10	30
**Random coil**	12	12	19	21	15	52	24
**β-Turns**	22	9	10	12	19	6	13
	RMSD from PDB	9	9	17	11	27	22

### Circular dichroism indicates stability rescue by chlorin e6

**Fig 5:**
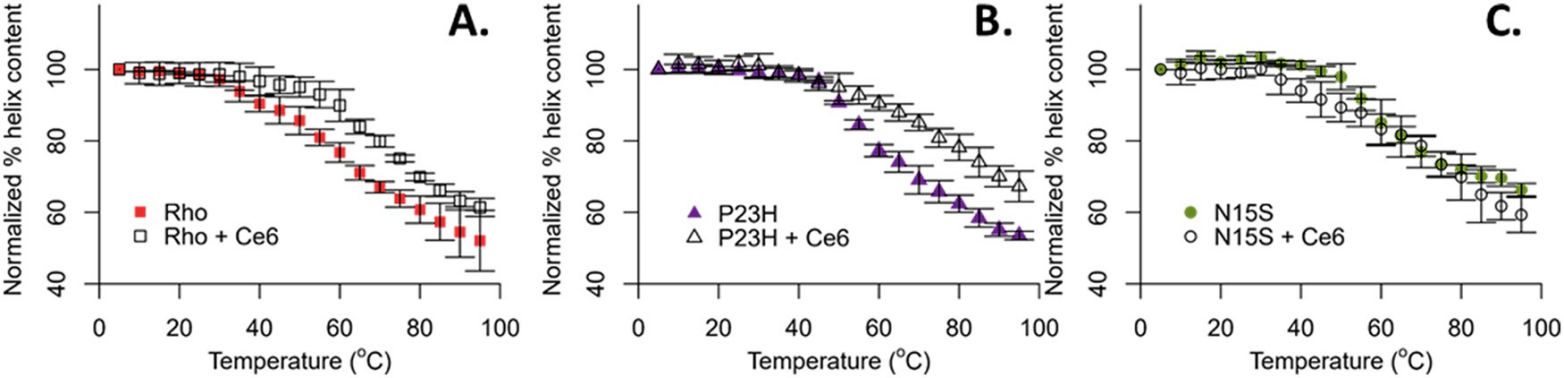
Circular dichroism spectroscopy of WT and mutant rhodopsin in the presence and absence of chlorin e6. Thermal denaturation studies of rhodopsin in the presence and absence of chlorin e6 (Ce6) were evaluated using circular dichroism (CD). **A.** CD melting spectra of WT rhodopsin (2.5 μM) with and without 100 μM Ce6. **B.** CD melting spectra of P23H. **C.** CD melting spectra of N15S. Concentrations of protein and chlorin e6 in B and C were the same as in A.

The FTIR results clearly showed a loss of α-helix structure when going from the WT to N15S and further loss when going from N15A to P23H. Previously, we showed that in WT, α-helix content can be stabilized by addition of chlorin e6 [21]. We therefore hypothesized that chlorin e6 may be particularly beneficial in stabilizing P23H. The effect of adding chlorin e6 to WT rhodopsin, P23H and N15S rhodopsin are shown in Fig 5A, 5B and 5C, respectively. One can see that similar to WT, the P23H mutant protein can be stabilized by chlorin e6, while the effect on N15S is smaller than both P23H and WT. We conclude that especially for P23H, addition of chlorin e6, may be a viable therapeutic avenue.

## Discussion

To better understand the molecular consequences of the N-terminal disease-causing mutations N15S and P23H, and how these may relate to the associated clinical phenotypes, we analysed these two mutations in detail *in vitro*, where we compared their structure, folding, stability, and glycosylation. We then compared their molecular behavior with the clinical-functional phenotype, in particular the variability in severity and the disease progression rates of patients carrying the N15S and P23H *RHO* mutations [1].

P23H and N15S proteins had both been shown previously to misfold [3,7]. Furthermore, trafficking of human N15S expressed in *Xenopus laevis* was localized both at the rod outer segments and as aggregated fractions in the Golgi membranes [3]. P23H is also found primarily in aggresomes [7,10,11,35–38]. Subcellular localization of P23H rhodopsin in cells is now even used to screen for pharmacological screening of small molecules to rescue P23H [39]. However, since these studies were carried out independently, a quantitative comparison was not possible. Here, we conducted all studies for P23H and N15S rhodopsin proteins in parallel with the WT, using COS-1 and HEK293 cells. We confirmed that the thermal instability of P23H was much higher, with a half-life about half of the N15S protein. Our estimated half-life values are virtually identical to those obtained independently by others [14]. Stability of the light-activated Meta II state on the other hand was found here and previously [14] to be similarly impaired for N15S and P23H, providing evidence for why the N15S leads to disease despite the otherwise mild effects on molecular function [1]. Furthermore, the proportion of unglycosylated P23H species was much higher than both WT and N15S. Thus, while our studies cannot provide an answer to the debate [3,17,18] on whether it is glycosylation or amino acid replacement or both that is responsible for the molecular consequences of the mutation, we can hereby quantitatively conclude that there are differences in the extent to which structure and stability is affected in the two mutations that aligns well with the severity of the respective phenotypes [1].

FTIR, which is a suitable tool for working out similarities [40] and dissimilarities of protein folds [41,42], suggests differences in secondary structure content between both, P23H and N15S, as compared to WT. Using this approach, we showed that N15S is more folded than the P23H rhodopsin. Recently, the opsin structures of P23H and of another ADRP *RHO* amino acid replacement, G188R, that we investigated previously [20,43] were characterized in cells by FTIR microspectroscopy [44]. Both, P23H and G188R were found aggregated in the ER where they exhibited elevated β-sheet (14% more as compared to WT) and reduced α-helical structure (8% less as compared to WT). The authors highlighted the need to “exclude secondary effects occurring in the ER that may contribute to the FTIR spectrum obtained from a cell” [44] by conducting FTIR studies with purified receptors, which is what we performed in this investigation. Our FTIR results indeed support the earlier conclusion from microspectroscopic FTIR studies on cells [44] that there is an increase in β-sheet and decrease in α-helical structure in proteins with ADRP mutations. This is also consistent with previous CD analysis of P23H that showed a decreased α-helical content in misfolded fractions as compared to WT [8]. The RMSD of all four structure elements determined shows that N15S in PBS is closer to WT than P23H.

Despite the fundamental differences in how the two mutant proteins behave *in vitro*, both displayed a dramatic, WT-like improvement in folding yields after addition to culture of either 9-*cis* or 11-*cis* retinal. These results emphasize the importance of 11-*cis* retinal in the biosynthesis and trafficking in the cells [9,13,14,45,46] and the therapeutic potential of vitamin A palmitate [12,47] or any other agent capable of promoting better rhodopsin folding *in vivo* in patients affected with these and other mutations leading to significant rhodopsin misfolding.

In addition, we propose here that a molecule capable of enhancing thermal stability of secondary structure in WT rhodopsin [21,23,24], chlorin e6, should be particularly suitable to rescue the destabilizing effects of P23H. We find that the effects of chlorin e6 on stability of P23H are more pronounced than on N15S.

In summary, our ability to quantitatively compare the characteristics of the two mutated proteins allowed us to study the extent of severity in molecular and functional terms. The molecular/biophysical *in vitro* studies of the purified proteins described here all suggest that the phenotype of N15S is significant but is milder than that of P23H. This is in line with previous predictions that P23 is located within the rhodopsin folding core (Fig 1, circled in red, folding core highlighted in blue), while N15 is close-by but not actually predicted to participate in the folding core [48].

## Supporting information

S1 File (DOCX)
