# Correction: Comparison of the molecular properties of retinitis pigmentosa P23H and N15S amino acid replacements in rhodopsin

**DOI:** 10.1371/journal.pone.0225153

**Published:** 2019-11-07

**Authors:** James Mitchell, Fernanda Balem, Kalyan Tirupula, David Man, Harpreet Kaur Dhiman, Naveena Yanamala, Julian Ollesch, Joan Planas-Iglesias, Barbara J. Jennings, Klaus Gerwert, Alessandro Iannaccone, Judith Klein-Seetharaman

The affiliation and email address for the eleventh author are incorrect. The current address for Alessandro Iannaccone should be listed as affiliation #5. Alessandro Iannaccone is affiliated with #4: Retinal Degeneration & Ophthalmic Genetics Service & Lions Visual Function Diagnostic Lab, Hamilton Eye Institute, Dept. Ophthalmology, University of Tennessee Health Science Center, Memphis, Tennessee, United States of America, and with #5: Center for Retinal Degenerations and Ophthalmic Genetic Diseases, Duke Eye Center, Dept. Ophthalmology, Duke University School of Medicine, Durham, North Carolina, United States of America. Dr. Iannaccone’s email address is alessandro.iannaccone@duke.edu.
