# Supplementary material for: Comparison of the molecular properties of retinitis pigmentosa P23H and N15S amino acid replacements in rhodopsin
Source: PLoS One. 2019 May 17;14(5):e0214639. doi: 10.1371/journal.pone.0214639 (PMC6524802; doi:10.1371/journal.pone.0214639)
Supplement: S1 File — (DOCX) [file pone.0214639.s001.docx]

Comparison of the Molecular Properties Retinitis Pigmentosa P23H and N15S Amino Acid Replacements in Rhodopsin

Supplementary Online Materials

Supplementary Methods

Construction of rhodopsin genes encoding amino acid replacements in pMT4 (for transient transfection)

Single amino acid replacements P23H and N15S were prepared by a two-step PCR mutagenesis technique using the synthetic bovine opsin gene in the expression vector pMT4 ([Oprian, Molday et al. 1987](#_ENREF_54)). The first step involved a PCR reaction to create two fragments for each mutation. One fragment was created using the upstream universal primer containing an EcoRI restriction site (underlined) 5’-TATGAATTCCACCATGAACGGGACCGAGGGGC-3’ and a second downstream primer specific for each of the single codon replacement. The second fragment was generated from the upstream primer specific for each codon replacement and the downstream universal primer that contains a NotI site (underlined): 5’-TATTGCGGCCGCTTAGGCAGGCGCCAGTTG3’. The following primers were used to create mutations (the desired mutation site is underlined): P23H: Upstream: 5’-GTGGTGCGCAGCCACTTCGAGGCT-3’ and Downstream: 5’-CGGAGCCTCGAAGTGGCTGCGCAC-3’; N15S: Upstream: 5’-GTTCCTTTCTCCTCCAAGACGGGC-3’ and Downstream: 5’-CACGCCCGTCTTGGAGGAGAAAGG-3’. The second step involved a PCR reaction to generate the fragments using the universal primers and templates created from the first step. The PCR products from the second step were used to replace the WT opsin gene in the pMT4 vector using EcoRI and NotI restriction sites. The final pMT4 vector with P23H or N15S replacements in the *RHO* genes were confirmed by DNA sequencing.

Construction of rhodopsin genes in pACMC-tetO (for generating inducible stable cell lines)

For stable cell line generation, P23H and N15S gene fragments from pMT4 were sub-cloned into the tetracycline inducible expression system vector pACMV-tetO, which was engineered to successfully express rhodopsin with WT sequence or carrying amino acid replacements ([Reeves, Kim et al. 2002](#_ENREF_58)). Unique KpnI and NotI sites in pACMV-tetO were utilized for sub-cloning. Since there was an internal KpnI site within the N15S and P23H opsin gene fragment in pMT4, we disrupted the internal KpnI site and introduced a new KpnI site upstream of the start codon. This goal was accomplished using QuikChange® Multi Site-Directed Mutagenesis Kit (Stratagene) and custom designed primers. Following this, the N15S and P23H gene fragments from pMT4 were obtained by KpnI/NotI restriction digestion and ligated into pACMV-tetO. The final cloning products containing the altered rhodopsin genes were confirmed by DNA sequencing.

COS-1 cell culture and transient transfection

Transient transfection of COS-1 cells was carried out as described ([Oprian, Molday et al. 1987](#_ENREF_54)), with the exception that the cells were harvested 72h after transfection. The cells were solubilized with 1% (w/v) DM (dodecyl maltoside) for one hour and the proteins were purified by 1D4 immunoaffinity chromatography in 0.05% DM as described ([Hwa, Reeves et al. 1999](#_ENREF_23)).

Establishment of stable cell lines

Tetracycline inducible HEK293S stable cell lines were established as described previously ([Reeves, Kim et al. 2002](#_ENREF_58)). Stable cell lines of WT, N15S and P23H were grown in complete media and when cells were about 80-90% confluent, they were washed twice with phosphate buffered saline (PBS) and induced with induction media: complete media with tetracycline (2µg/ml) and sodium butyrate (5mM). At 2 and 24 hours after induction, *9-cis* retinal or *11-cis* retinal (chromophore) was added directly to the cells at 10µM concentration so that the final concentration of the chromophore is 20µM ([Krebs, Holden et al. 2010](#_ENREF_39)). The chromophore was added in dark and all subsequent steps were also performed in the dark. Harvesting of the cells was done 48 hours after induction. Proteins were solubilized in DM and purified by ID4 immunoaffinity chromatography in 0.05% DM as described ([Hwa, Reeves et al. 1999](#_ENREF_23)).

Protein purification

After solubilization of the cells, the suspension was centrifuged for 30 min at 35,000 rpm and 4^0^C. The supernatant was mixed with 1D4 Sepharose beads (approximate binding capacity of 1 μg rhodopsin/ μl of resin) for at least 6 h at 4^0^C. The resin was then washed with 50 bed volumes of 0.05% (w/v) DM in PBS followed by 10 bed volumes of 0.05% (w/v) DM in 2 mM Na_2_HPO_4_/NaH_2_PO_4_ (pH 6.0). WT, P23H and N15S proteins were eluted with 70μM C-terminal nonapeptide (TETSQVAPA) in 0.05% (w/v) DM in 2 mM Na_2_HPO_4_/NaH_2_PO_4_ (pH 6.0) or PBS as indicated.

UV-Visible absorption spectroscopy

UV-visible absorption was recorded by using a UV spectrophotometer (Perkin–Elmer λ 25), equipped with a multiple water-jacketed cuvette holders connected to a circular water bath, which controlled the temperature. Molar extinction value used for rhodopsin was 40,600
M^-1^cm^-1^. Thermal denaturation experiments of purified WT, P23H and N15S proteins reconstituted with 9-*cis* retinal in 0.05% (w/v) DM in 2 mM Na_2_HPO_4_/NaH_2_PO_4_ (pH 6.0) were performed either at 55^o^C with a bandwidth of 2nm, response time of 1s, and scan speed of 240nm/min (data not shown due to rapid decay) or at 37^o^C in the 250-650 nm range, using a 10mm path length cell, bandwidth of 1nm, response time of 1s and scan speed of 960nm/min. Thermal stability was investigated by following the loss of 500nm absorbance as a function of time at 37^o^C. The decrease in absorbance was expressed as percent of initial absorbance (at t=0).

Deglycosylation with PNGase F

For cleavage of oligosaccharide chains from rhodopsin glycosylation sites, 1μg of WT, P23H or N15S rhodopsin reconstituted with 11-*cis* retinal and purified in 0.05% (w/v) DM in PBS (pH 7.2) and 125 units of peptide: N-glycosidase F (PNGase F, Sigma) were incubated for 3 hrs at room temperature in 20 mM Tris-Cl pH 8.0, 0.05% (w/v) DM, 0.5% SDS, 5 mM EDTA, and 0.1 mM PMSF. This enzyme cleaves the link between asparagine and N-acetylglucosamines. Glycosylation was assessed before (–) and after (+) treatment with PNGase F.

Immunoblotting

Protein samples were resolved by SDS-PAGE on 15% polyacrylamide-Tris vertical slab gels and transferred onto nitrocellulose membrane (Bio-Rad) according to standard protocols. Membrane was blocked overnight at 4^0^C in blocking solution containing 2% (w/v) bovine serum albumin (sigma), 5% (w/v) non-fat dry milk (Bio-Rd) and 0.1% Tween 20 in PBS. 1D4 primary antibody was diluted in blocking buffer and incubated with the membrane for an hour at room temperature. Membrane was washed in PBS three times and further incubated with horseradish peroxidase-conjugated anti-mouse secondary antibodies (Bio-Rad) in 1:3000 dilution for an hour at room temperature. The protein signals were visualized using SuperSignal West Pico Chemiluminescent Substrate kit (Pierce) following the protocol provided by the supplier.

Fluorescence spectroscopy

Meta II fluorescence of WT, P23H and N15S rhodopsin was measured as described ([Farrens and Khorana 1995](#_ENREF_15)). The fluorescence was recorded using a Varian Cary Eclipse instrument. Excitation and emission wavelengths for the recording were 295 and 330nm, respectively. To acquire the spectra, a slit width of 5 nm for excitation and 10 nm for emission were used. Samples were placed in the cuvette holder for 10min for equilibration, followed by 10min of dark reading prior to illumination with yellow light. The experiments were performed using 0.5µM purified rhodopsin reconstituted with 9-*cis* retinal in 0.05% (w/v) DM in 2 mM Na_2_HPO_4_/NaH_2_PO_4_ (pH 6.0). In order to calculate Meta II half-lives, the data was analyzed by fitting to single and double component non-linear regression functions using Sigmaplot 10.0 scientific graphing software and R.

Fourier transform infrared (FTIR) spectroscopy

FTIR spectra were collected on a Bruker IFS 88 FTIR-spectrometer equipped with a 7 reflexion diamond µATR sensor (predecessor of DuraSamplIR II, Smiths Detection, UK). 256 full interferograms were acquired in both mirror directions with a 2 cm-1 resolution and 4fold zero filling before Fourier transformation with Blackman-Harris-3-term apodization and Mertz phase correction. 10 µl purified protein solution (PBS buffer plus 0.05% DM either in H_2_0 or D_2_0) of WT, N15S and P23H (all reconstituted with 11-cis retinal) were dried in a gentle nitrogen stream on the ATR sensor surface to form a stable protein/lipid film. A remaining hydration by tightly bound water molecules was identified spectrally. H/D-Exchange was achieved by the addition of 10 µl D_2_O to the sample film and redrying in 85 % rel. humidity D_2_O saturated N_2_ atmosphere.

To estimate the protein secondary structure fractions, a calibrated amide I band decomposition was performed as described previously ([Ollesch, Kunnemann et al. 2007](#_ENREF_52)). Briefly, amide I bands were decomposed into a set of Cauchy curves to obtain a resulting set that showed (i) a high quality band decomposition for all bands and (ii) has a minimum deviation of the WT secondary structure fractions from calibration data. This was obtained from the X-ray crystal structure of Rhodopsin (PDBID: 1L9H) using a STRIDE secondary structure analysis ([Frishman and Argos 1995](#_ENREF_16)). Missing residues in the PDB file were attributed to random coil. Two respective sets of initialization parameters needed as input for the decomposition were determined to fulfill above criteria (i) and (ii) for the protonated and for the deuterated samples. Note that identical initialization parameters do not determine identical results, as seen in Figure 4 E. To deconvolute this spectrum, the highest frequency β-sheet component was given to, but eliminated by the curve fitting algorithm. The relative contents of different secondary structure fractions were estimated as the ratio between the band integrals corresponding to specific secondary structure fractions and the whole area of the amide I band region.

Circular Dichroism (CD) Spectroscopy.

CD spectra were acquired using a JASCO J‐810 spectropolarimeter. All spectra were recorded from 195 to 260 nm, using a bandwidth of 1 nm, scan speed of 100 nm min^−1^, and time constant of 1 s. An average of 10 scans was acquired for each spectrum. Reference spectra were subtracted from the subsequently recorded samples containing protein. We did not correct for the presence of nonapeptide in rhodopsin samples, as we estimate its contribution to the ellipticity at 222 nm to be less than 5%. The thermal denaturation of rhodopsin in the presence of Ce6 were recorded by increasing the temperature from 5 to 100°C, with intervals of 5°C. Rhodopsin and Ce6 concentrations used were 2.5 and 100 μM, respectively. The samples were placed in a 1 mm quartz cell. Qualitative estimates of helix content were obtained from the CD spectra by spectral deconvolution using CDPro software [32,33]. Protein reference set 10, which includes membrane proteins, was used.

Supplementary Results

FTIR Spectroscopy

The results presented correspond to deconvoluted FTIR spectra in the amide I region (1600 - 1700 cm^-1^) with center around 1650 cm^-1^ corresponding mainly to C=O stretching of the peptide backbone. The percentages of the secondary structures are estimated by integrating the intensities for each secondary structure element and expressed as a fraction of the total amide I region. In WT, the content of α-helix, β-sheet, random coil and turns in aqueous conditions were 63%, 16%, 12% and 9%, respectively. The observed estimates are in good agreement with the estimated secondary structure content from the X-ray crystal structure of rhodopsin (9 % root mean squared deviation). The theoretical secondary structure distributions obtained using the rhodopsin crystal structure with Protein Databank identifier 1L9H gives 64%, 3%, 12% and 22% are calculated for α-helix, β-sheet, random coil and turns, respectively. In the case of N15S rhodopsin, a decrease in α-helix content (42%), and an increase in β-sheet (25%), random coil (21%) and turns (12%) was observed when compared to WT. The P23H rhodopsin exhibited even more severe structural defects when compared to WT: a strong increase in the random coil fraction (52%) was observed, and the α-helix, β-sheet, and turn fractions decreased to 32%, 10%, and 6%, respectively. These results indicate that the structure of N15S is more folded than the P23H rhodopsin.

Since the bands corresponding to α-helix, random coil and β-sheet severely overlap and are difficult to separate under aqueous condition, ATR-FTIR spectra of the samples were recorded in D_2_O. Backbone H/D exchange leads to shifts in the bands corresponding to secondary structure elements in the amide I region, with bands originating from helices shifting less than random coil bands resulting in a skewed amide I band (Main Manuscript, **Figure 2D-F**). The re-estimated secondary structure analysis of WT rhodopsin showed a decrease in α-helix (59%), and β-sheet (12%) and an increase in random coil (19%) and turns (10%). An increase in α-helix (48%), and turns (19%) and a decrease in β-sheet (18%) and random coil (15%) fraction was observed for N15S, and an increase in β-sheet (30%) and turns (13%), a decrease in random coil (24%) and a similar fraction of α-helical fraction (33%) was observed for P23H. These results indicate that the investigated structural features of N15S rhodopsin were more similar to WT rhodopsin than those of P23H rhodopsin. The overall increase in β-sheet and random coil fractions and a decrease in α-helix content support the conclusion that P23H is less folded as compared to N15S and WT. Further, an increase of 18% in β-sheet content compared to WT (30% vs 12%) was observed for P23H.

Deglycosylation analysis

Table A. Center of the positions of monomer bands shown in Figure 3F.

| Lane | peak | mean MW | sd MW | mean % | sd % |
| --- | --- | --- | --- | --- | --- |
| WT minus | peak 1 | 37 | 1.0 | 96.48 | 7.04 |
| WT plus | peak 1 | 31 | 0.9 | 100 | 0 |
| P23H minus | peak 1 | 29 | 1.7 | 21.88 | 10.24 |
| P23H minus | peak 2 | 35 | 1.6 | 78.12 | 10.24 |
| P23H plus | peak 1 | 32 | 1.6 | 89.59 | 20.82 |
| N15S minus | peak 1 | 33 | 2.9 | 50.82 | 4.63 |
| N15S minus | peak 1 | 43 | 3.3 | 49.18 | 4.63 |
| N15S plus | peak 1 | 30 | 2.3 | 41.57 | 6.31 |
| N15S plus | peak 2 | 36 | 5.6 | 58.43 | 6.31 |
